# TME7/366: Borderless Teleradiology with CHILI

**DOI:** 10.2196/jmir.1.suppl1.e114

**Published:** 1999-09-19

**Authors:** U Engelmann, A Schroeter, M Schwab, U Eisenmann, H P. Meinzer

**Affiliations:** ^1^German Cancer Research CenterHeidelbergGermany

**Keywords:** Teleradiology, PACS, Privacy, Teleconferencing, CHILI

## Abstract

**Introduction:**

The CHILI software family started as a dedicated teleradiology system, known under the name MEDICUS. The second generation teleradiology system CHILI has then been designed to match the teleradiology requirements of the ACR and the needs of the MEDICUS users. The experience of software developers and teleradiology users of the first years of clinical use have been integrated into the new design which started in 1996. This paper describes the general system design and applications areas.

**Methods:**

The system is based on a component based architecture. The most powerful communication protocol for data exchange and teleconferencing is the CHILI protocol which includes a strong data security concept. This includes all measures which are necessary to comply with German and European requirements and law. But it cannot be expected that all communication partners have the same teleradiology system. CHILI offers additional communication Methods:

1. The DICOM protocol allows the exchange of images with all other DICOM compliant systems.
2. Classical e-mail (SMTP protocol) can be used to send images DICOM or JPEG encoded.
3. Remote copy functions from host to host.
4. File transfer protocol (ftp).

These transfer methods enable the CHILI user to send images nearly to everybody with a computer and a network. Drawbacks are that teleconferences are not possible and that the user has to take reasonable precautions for data privacy and security. As users do always need more functionality than a system can provide, we designed the CHILI PlugIn mechanism. Users can extend the system by powerful image post-processing functions or interfaces to other information systems. PlugIns can be existing programs or be programmed with interfaces to the CHILI components. The developer is free in his choice of programming languages and interface toolkits.

**Results:**

The CHILI architecture is a powerful and flexible environment for PACS and teleradiology. More than 40 systems are currently running in clinical routine in Germany. More than 250 thousand images have been distributed between the communication partners in the last two years. The feedback and suggestions of the users influenced the system architecture by a great extent.

**Discussion:**

Current limitations of teleradiology are still vendor dependent communications protocols. DICOM is a start for platform independence. But it still lacks many aspects, such as teleconferencing or data security and privacy. The proposed and implemented systems tries to be as platform independent, open, and as secure as possible.

